# Recent Advances in Understanding of Pathogenesis of Alcohol-Associated Liver Disease

**DOI:** 10.1146/annurev-pathmechdis-031521-030435

**Published:** 2022-10-21

**Authors:** Xiaoqin Wu, Xiude Fan, Tatsunori Miyata, Adam Kim, Christina K. Cajigas-Du Ross, Semanti Ray, Emily Huang, Moyinoluwa Taiwo, Rakesh Arya, Jianguo Wu, Laura E. Nagy

**Affiliations:** 1 Northern Ohio Alcohol Center, Department of Inflammation and Immunity, Cleveland Clinic, Cleveland, Ohio, USA; 2 Department of Gastroenterology and Hepatology, Cleveland Clinic, Cleveland, Ohio, USA; 3 Department of Molecular Medicine, Case Western Reserve University, Cleveland, Ohio, USA

**Keywords:** alcohol-associated liver disease, ALD, programmed cell death, PCD, pattern recognition receptors, PRR, complement, gut-liver axis

## Abstract

Alcohol-associated liver disease (ALD) is one of the major diseases arising from chronic alcohol consumption and is one of the most common causes of liver-related morbidity and mortality. ALD includes asymptomatic liver steatosis, fibrosis, cirrhosis, and alcohol-associated hepatitis and its complications. The progression of ALD involves complex cell-cell and organ-organ interactions. We focus on the impact of alcohol on dysregulation of homeostatic mechanisms and regulation of injury and repair in the liver. In particular, we discuss recent advances in understanding the disruption of balance between programmed cell death and prosurvival pathways, such as autophagy and membrane trafficking, in the pathogenesis of ALD. We also summarize current understanding of innate immune responses, liver sinusoidal endothelial cell dysfunction and hepatic stellate cell activation, and gut-liver and adipose-liver cross talk in response to ethanol. In addition, we describe the current potential therapeutic targets and clinical trials aimed at alleviating hepatocyte injury, reducing inflammatory responses, and targeting gut microbiota, for the treatment of ALD.

## INTRODUCTION

Lifestyle, including diet, has an important impact on the development of multiple diseases. Excessive alcohol consumption is one controllable lifestyle factor known to contribute to tissue injury and to a number of diseases. Worldwide, 3 million deaths every year result from harmful use of alcohol, representing 5.3% of all deaths (1). Alcohol-associated liver disease (ALD) is one of the major diseases arising from chronic, heavy alcohol consumption and is one of the most common causes of liver-related morbidity and mortality (2, 3). In 2010, the worldwide rate of alcohol-attributable cirrhosis death was 7.2 deaths per 100,000 people (4.6 in females and 9.7 in males) (3). In 2019 in the United States, 53,486 deaths from liver disease occurred in males, with 45.6% involving alcohol, and 32,202 deaths from liver disease occurred in females, 39.0% of which were alcohol related. Importantly, ALD is currently a major indication for liver transplantation in the United States and Europe, as the incidence of hepatitis C is declining (3, 4).

ALD encompasses a spectrum of diseases including asymptomatic liver steatosis, fibrosis, cirrhosis, and alcohol-associated hepatitis (AH) and its complications. Approximately 8–20% of chronic heavy drinkers will develop alcohol-related cirrhosis and, of these patients, approximately 2% will develop hepatocellular carcinoma (2). ALD, which progresses from fatty liver, as the initial stage, to fibrosis and AH, accounts for approximately half of the causes of nonviral cirrhosis, particularly as viral hepatitis is waning, and is also major cause of nonviral liver cancer. Genetic polymorphisms of alcohol-metabolizing enzymes are associated with heavy drinking habits and dependence, while genetic polymorphisms such as patatin-like phospholipase encoding 3 (*PNPLA3*) are risk factors for fatty liver formation and progression of liver pathology (5). However, the specific genes involved in the progression of ALD or its complications are not well studied. In addition to cellular damage caused by reactive oxygen species (ROS) generated during alcohol metabolism, changes in the gut microbiota (dysbiosis) and the immune response to these changes play major roles in the development and progression of ALD (6). In this review, we focus on the impact of chronic alcohol on dysregulation of homeostatic mechanisms regulating injury and repair in the liver. In particular, we discuss recent advances in understanding disruption of pathways regulating cell death and survival, innate immune responses, and organ-organ interactions in ALD. We also summarize the current potential therapeutic targets and clinical trials for the treatment of ALD.

## ALCOHOL METABOLISM

Alcohol metabolism occurs via both oxidative pathways, involving alcohol dehydrogenases (ADHs), microsomal cytochrome P450 enzymes (CYPs), or peroxisomal catalase, and nonoxidative pathways. ADHs, a class of zinc dehydrogenases residing in the cytosol, catalyze the oxidation of primary and secondary alcohols to their corresponding aldehydes or ketones with the reduction of NAD^+^ to NADH and can also catalyze the reverse reaction. Human ADHs are encoded by at least seven genes on chromosome 4, divided into five classes, and differentially distributed in tissues. Most classes of ADHs exhibit the highest activity in the liver. The class I ADHs (ADH1A, ADH1B, and ADH1C) carry out oxidation of most of the ingested alcohol (ethanol) in the liver, generating acetaldehyde (7).

Acetaldehyde is highly reactive and forms adducts with cellular proteins, nucleic acids, and lipids, compromising normal cellular functions and underlying alcohol’s pathogenicity. Acetaldehyde also competes with other endogenous aldehydes originating from the metabolism of physiological chemicals such as dopamine, norepinephrine, and serotonin. Once generated, the toxic acetaldehyde is quickly converted to less toxic acetate by aldehyde dehydrogenases (ALDHs), and the acetate is broken down into water and CO_2_ for easy elimination.

Chronic alcohol consumption increases the expression and activity of some CYPs, particularly CYP2E1, an isoform that is conserved across mammalian species and expressed in multiple cell types and tissues. CYP2E1 assumes a significant role in the oxidation of ethanol to acetaldehyde, especially in the presence of high concentrations of ethanol (K_m_ = 8 to 10 mM, compared with 0.2 to 2.0 mM of hepatic ADH). It is also considered a leaky cytochrome, generating excess ROS even in the absence of substrate (8). Catalase is a heme-containing enzyme found in all living organisms and particularly expressed in the peroxisomes. Catalase normally decomposes H_2_O_2_ to H_2_O and O_2_ in the presence of electron donors. Ethanol, as an electron donor, is thus oxidized to acetaldehyde by catalase (8). Although catalase and CYP2E1 play a much smaller role in alcohol oxidation compared with ADHs, they significantly impact the rate of oxidation of ethanol to acetaldehyde in the brain, where ADH activity is low (9).

Several nonoxidative pathways of alcohol metabolism result in the enzymatic conjugation of ethanol to endogenous metabolites to produce nonoxidative ethanol end products such as ethyl glucuronide, ethyl sulfate, phosphatidylethanol, and fatty acid ethyl ester. Despite accounting for an overall low fraction of total ethanol metabolism, the resulting ethanol metabolites can be useful as retrospective biomarkers in assessing ethanol intake due to their relatively slower elimination rates (10).

In summary, the generation of highly reactive acetaldehyde and excess ROS during oxidative ethanol metabolism leads to cellular injury, particularly in hepatocytes. The hepatic response to the accumulation of damaged cellular proteins, nucleic acids, and lipids impairs cellular functions and stimulates the induction of pathways, such as the unfolded protein response or the endoplasmic reticulum (ER) stress response. If the hepatocyte response to stress is insufficient, multiple pathways of cell death are activated.

## CELL DEATH AND PROSURVIVAL PATHWAYS

**Figure 1:**
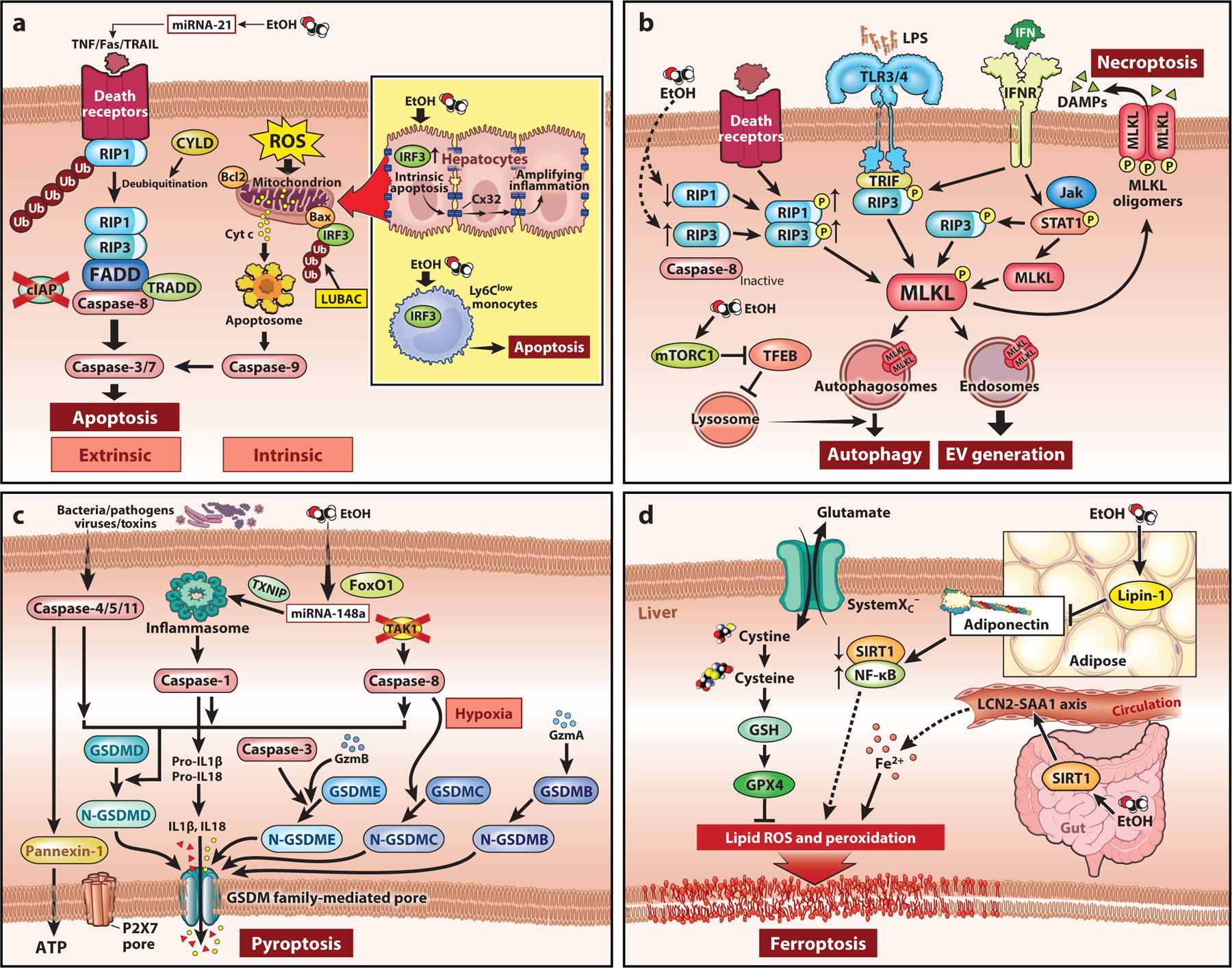
Multiple formats of programmed cell death in alcohol-associated liver disease. (*a*) Ethanol exposure induces Fas ligand and DR5-mediated extrinsic apoptotic pathways through miRNA-21. Activation of IRF3 initiates alcohol-induced hepatocyte apoptosis. cGAS-driven IRF3 signaling spreads through hepatic gap junction communication between hepatocytes via Cx32, thereby amplifying inflammation and accelerating hepatocyte apoptosis; ethanol also induces IRF3-mediated apoptosis in the Ly6C^low^ population. (*b*) Necroptosis classically depends on the death receptor action and RIP3-mediated phosphorylation of MLKL. In addition, TLRs and interferon receptors can induce activation of MLKL. Ethanol feeding induces phosphorylation of RIP1 and RIP3. In an acute-on-chronic model, ethanol impairs TFEB-mediated lysosome biogenesis through activation of mTORC1, resulting in insufficient autophagy. MLKL also plays a critical role in the regulation of endosomal trafficking and generation of EVs. (*c*) Ethanol can induce caspase-1-mediated pyroptosis via miRNA-148a-targeted overexpression of TXNIP in hepatocytes. The NLRP3 inflammasome pathway is activated in hepatocytes in response to LPS-induced ER stress. After the transition from chronic alcoholic steatohepatitis to alcohol-associated hepatitis, noncanonical caspase-112013GSDMD signaling is activated. (*d*) Ethanol feeding results in iron-dependent ferroptotic cell death, which is characterized by excessive accumulation of intracellular lipid ROS and lipid peroxidation. Adipose-specific overexpression of lipin-1 exacerbates steatosis, hepatobiliary damage, and mild fibrotic injury by a GPX4-independent induction of hepatic iron overload lipid peroxidation. Further, intestinal SIRT1 could mediate ethanol-induced hepatic iron metabolism dysfunction and ferroptosis through the circulating LCN2-SAA1 axis in a GPX4-independent mechanism, ultimately contributing to ethanol-induced liver injury. Solid arrows indicate direct promotion, dashed arrows indicate indirect promotion, and blunt-ended arrows indicate direct inhibition. Thin arrows pointing up or down next to items indicate increase or decrease. Abbreviations: Cx32, connexin 32; DR5, death receptor 5; ER, endoplasmic reticulum; EtOH, ethanol; EV, extracellular vesicle; GSDM, gasdermin; IFN, interferon; IFNR, interferon receptor; IRF3, interferon regulatory factor 3; LPS, lipopolysaccharide; miRNA-21, microRNA-21; NLRP3, NLR family pyrin domain containing 3; RIP3, receptor interacting protein kinase 3; ROS, reactive oxygen species; TFEB, transcription factor EB; TLR, Toll-like receptor. Adapted with permission, Cleveland Clinic Foundation ©2022. All rights reserved.

The intricate balance between prosurvival and death pathways of parenchymal and nonparenchymal cells is critical for regulating liver injury and inflammation during the progression of ALD (6). Programmed cell death (PCD) is a crucial and active process, serving to maintain tissue homeostasis in multicellular organisms. There are four major modes of PCD (Figure 1): apoptosis, necroptosis, pyroptosis, and ferroptosis (11). Recently, a newly recognized pathway for proinflammatory PCD called PANoptosis that is activated by bacterial and viral triggers was determined to be controlled by a multimeric protein complex—the PANoptosome. The PANoptosome can in parallel engage pyroptosis, apoptosis, and necroptosis (12). Additionally, autophagy and membrane trafficking, which share common components and affect each other, play an important role in repair of cell damage and cell survival (13). Dysregulation or hyperactivation of autophagy and membrane trafficking is associated with cell injury and death and contributes to the development of ALD (14–16). Hepatocellular fate in response to extracellular signaling depends on the cellular environment. Alcohol induces cellular oxidative and/or ER stress through ethanol metabolism, resulting in increased exposure to damage-associated molecular patterns (DAMPs). The liver is also exposed to gastrointestinal-derived pathogen-associated molecular patterns (PAMPs) due to the impact of ethanol on gut integrity. Cumulatively, these ethanol-induced insults result in activation of different cell death pathways. Here, we summarize the current understanding of the regulation of cell death and prosurvival pathways in the pathogenesis of ALD in multiple scenarios.

### Apoptosis

The extrinsic apoptotic pathway is typically activated by members of the tumor necrosis factor (TNF) family of death receptor (DR) ligands, comprising TNF, Fas ligand, and TNF-related apoptosis-inducing ligand (TRAIL) (11). The intrinsic pathway is commonly triggered via members of the B cell lymphoma 2 (Bcl-2) family, which control mitochondrial outer membrane permeabilization, cytochrome c release, and, subsequently, caspase activation (11). Francis et al. (17) previously reported that ethanol exposure induced Fas ligand and DR5-mediated extrinsic apoptotic pathways through microRNA-21 (miRNA-21). Recent studies have been focused on the retinoic acid-inducible gene I (RIG-I)-like receptor (RLR)-induced interferon regulatory factor 3 (IRF3)-mediated pathway of apoptosis (RIPA). In the RIPA branch, IRF3 is activated by LUBAC-mediated linear ubiquitination, which triggers its interaction with BAX to cause mitochondrial activation and apoptotic cell death (18). Szabo and colleagues (19) found that activation of IRF3 initiates alcohol-induced hepatocyte apoptosis, which fuels a robust secondary inflammatory response that drives ALD. Importantly, cGAS-driven IRF3 signaling spreads through hepatic gap junction communication between hepatocytes via connexin 32 (Cx32), thereby amplifying inflammation and accelerating hepatocyte apoptosis and, subsequently, damage (20). Additionally, hepatic monocyte/macrophage apoptosis also contributes to liver homeostasis and response to ethanol-induced damage. Lotersztajn and colleagues (21) revealed that interleukin 10 (IL-10) released from M2 Kupffer cells (KCs) promoted M1 KC death via apoptosis, to protect against alcohol-induced inflammation and injury. Sanz-Garcia et al. (22) demonstrated that *Irf3*^−*/*−^ mice were protected from Gao-binge ethanol-induced liver injury, associated with the suppression of ethanol-induced apoptosis in the Ly6C^low^ population (restorative). On the basis of these findings (Figure 1a), it is plausible that intervention in the apoptotic death pathway is a potential strategy to prevent alcohol-induced injury. However, genetic [*Bid*^−*/*−^ (23) or *Caspase-8*^−*/*−^ (24)] or pharmacological (23) (VX166, a pan-caspase inhibitor) inhibition of apoptosis is not completely protective in murine models of early ALD. These data suggest that cell-specific and/or additional forms of PCD are critical during progression of ALD.

### Necroptosis

Necroptosis classically depends on the phosphorylation of receptor interacting protein kinase 3 (RIP3) by RIP1. RIP3 then phosphorylates the critical effector MLKL, leading to its translocation to the plasma membrane, where it oligomerizes and forms pores that trigger necroptotic cell death (25). In addition to death receptors, other receptors, for example, Toll-like receptors (TLRs) and interferon (IFN) receptors, also induce MLKL-mediated necroptosis. Importantly, multiple studies have identified differential contributions of the RIP1-RIP3-MLKL axis to disease progression in murine models of ALD (mALD) (Figure 1b). Chronic ethanol feeding induces RIP3 expression in mouse livers and primary hepatocytes, while the mRNA and protein levels of RIP1 are markedly decreased by Gao-binge (acute-on-chronic) alcohol exposure. Inhibition of RIP1 by necrostatin-1 attenuates alcohol-mediated inflammation but not hepatocyte injury (26), while *Rip3*^−*/*−^ mice are protected from chronic ethanol-induced liver injury (26, 27). These data suggest that RIP3 likely contributes to the progression of ALD in a RIP1-independent mechanism. Indeed, casein kinase family members directly phosphorylate RIPK3 to activate necroptosis, likely interacting with RIP3 through its RHIM domain (28). *Mlkl*^−*/*−^ mice are only partially protected from Gao-binge and chronic ethanol-induced liver injury (29), suggesting that RIP3 and MLKL also likely function via independent, noncanonical mechanisms in mALD. Further study is needed to determine the underlying mechanism for regulating both the canonical and noncanonical functions, as well as domain-specific functions, of the RIP1-RIP3-MLKL axis in the context of alcohol exposure.

Of translational significance, circulating concentrations of RIP1 and RIP3 distinguish patients with AH from healthy controls (HCs), as well as from patients with nonalcoholic steatohepatitis (NASH). RIP3, but not RIP1, is likely a promising biomarker to predict prognosis in AH after diagnosis (29). These data are consistent with reports that circulating concentrations of cytokeratin-18 (M65) versus its caspase-3-mediated cleavage product (M30), released by necrotic/necroptotic versus apoptotic cell death, respectively, are also diagnostic and prognostic indicators in patients with AH (30, 31).

### Pyroptosis

The canonical pathway of pyroptotic cell death requires the priming and assembly of multiple signals, triggered by multiple DAMPs and PAMPs through TLRs. Signal integration is accomplished by the assembly of cytosolic pattern recognition receptors (PRRs), including nucleotide oligomerization domain (NOD)-like receptor (NLR) family pyrin domain containing 1 (NLRP1), NLRP3, NLRC4, absent in melanoma 2 (AIM2), and pyrin, and activation of caspase-1. Noncanonical pyroptosis is driven by caspase-4 and caspase-5 (human) or caspase-11 (mouse) (32). Upon activation, these caspases cleave gasdermin D (GSDMD), which then binds to lipids at the plasma membrane and forms oligomeric pores, thereby leading to pyroptosis. Meanwhile, activated caspase-1 controls the maturation of IL-1β and IL-18. The activated caspases (caspase-4, -5, and -11) also cleave pannexin-1, inducing ATP release and P2X7R-related pyroptotic cell death. In addition to GSDMD, pyroptosis can also be triggered by other members of the GSDM family. Active caspase-3 and caspase-8 can cleave GSDME and GSDMD, respectively. In addition, under hypoxic conditions, programmed death-ligand 1 (PDL1) translocates to the nucleus and regulates the transcription of GSDMC together with phosphorylated signal transducer and activator of transcription 3 (p-STAT3), resulting in the conversion of apoptosis to pyroptosis after TNF-α activates caspase-8. In the granzyme (Gzm)-mediated pathway, GzmA and GzmB in cytotoxic lymphocytes enter target cells through perforin and induce pyroptosis. GzmA hydrolyzes GSDMB, and GzmB, in turn, directly activates GSDME (32).

There is a growing body of evidence demonstrating that GSDM family–mediated pyroptosis is a key driver of ALD in both patients and animal models (Figure 1c). Previously, *Tlr4*^−*/*−^ mice were shown to be protected from early alcohol-induced injury (33). Heo and colleagues (34) found that ethanol induces caspase-1-mediated pyroptosis via miRNA-148a-targeted overexpression of TXNIP in hepatocytes. The NLRP3 inflammasome pathway is activated in hepatocytes in response to lipopolysaccharide (LPS)-induced ER stress. Metabolically derived DAMPs, including ATP and soluble uric acid, released from damaged hepatocytes in response to alcohol binge, trigger the production of inflammasome-dependent IL-1β from immune cells. *Nlrp3*-deficient mice were resistant to alcohol-induced inflammation and injury (35). Consistent with these observations, inhibition of ATP or uric acid prevents inflammasome activation and IL-1β production, thereby protecting mice from ethanol-induced damage (36). Pharmacological inhibition of IL-1β/IL-1R1 signaling by recombinant human IL-1R antagonist attenuated alcohol-induced liver inflammation, steatosis, and damage (37). Khanova et al. (38) reported that, after the transition from chronic alcohol-associated steatosis to AH, activation of noncanonical caspase-11-GSDMD signaling, but not canonical caspase-1-IL-1β signaling, was evident in livers from both murine models of ALD and patients with AH. *Caspase-11* deficiency prevented ethanol-mediated GSDMD activation, hepatocellular death, and bacterial burden.

### Ferroptosis

Ethanol feeding results in iron-dependent ferroptotic cell death, which is characterized by excessive accumulation of intracellular lipid ROS and consequent lipid peroxidation resulting from depletion of iron-dependent glutathione and inactivation of glutathione peroxidase 4 (GPX4) (39). As *Gpx4*^−*/*−^ mice exhibit early embryonic lethality (40), multiple chemical compounds, targeting either GPX4 or other regulators of ferroptosis, are broadly used to elucidate the role of ferroptosis in multiple disorders (41). For instance, ferrostatin-1, which serves as a lipid ROS scavenger, notably ameliorates ethanol-induced hepatocellular injury, both in vitro and in vivo (42). Ferroptosis is also implicated in the progression of ALD through the liver-gut axis and the liver-adipose axis (Figure 1d). Zhou et al. (43) first found that adipose-specific overexpression of lipin-1 exacerbates steatosis and hepatobiliary damage and leads to mild fibrotic injury by a GPX4-independent induction of hepatic iron overload lipid peroxidation. In this scenario, they speculated that alternative mechanisms might be at play in parallel with the regulation of ferroptosis by GPX4. Further, they reported that intestinal SIRT1 was also required for ethanol-induced dysfunction of hepatic iron metabolism and ferroptosis. This pathway involved changes in the circulating LCN2-SAA1 axis in a GPX4-independent mechanism, ultimately contributing to ethanol-induced liver injury (44). In summary, dampening hepatic ferroptosis signaling may have a therapeutic potential for preventing mALD. The current data suggest that targeting ferroptosis will likely involve a better understanding of the cross talk between liver and other organs, such as adipose and gut. Further, while these initial studies characterizing ferroptosis have focused on describing characteristic hallmarks of this pathway of cell death, very little is known regarding the precise mechanisms that drive the ferroptotic cascade downstream of lipid peroxidation. For example, it is not clear whether intestinal microbiota or metabolites migrating into the liver through the portal vein can trigger hepatic ferroptosis in ALD.

### Autophagy and Membrane Trafficking

The involvement of hepatocyte autophagy in ALD is important and complex. Acute alcohol consumption activates autophagy (45), whereas chronic ethanol exposure decreases lysosome-mediated lipid droplet turnover in hepatocytes through inactivation of Rab7 (46) or reduced dynamin 2 activity (47). In both acute and acute-on-chronic models of ethanol exposure, ethanol impairs transcription factor EB (TFEB)-mediated lysosome biogenesis through activation of mTORC1, resulting in impaired/insufficient autophagy (16). Furthermore, dysregulated autophagy and lysosome function have been linked to exosome production in ALD (48). The total number of circulating extracellular vesicles (EVs) was increased in patients with AH and in mice after chronic ethanol feeding (49, 50). Studies have shown that the EV cargo, miR-192 and miR-30a, as well as other proteins including heat shock protein 90 and CD40 ligand, are potential biomarkers and mediators of ethanol-induced injury (49–51).

There are considerable interactions between autophagy and membrane trafficking and different forms of PCD. Autophagy is considered as an early adaptive response to injury that occurs prior to apoptosis; however, hyperactivation of autophagy also results in apoptotic cell death through common regulators, such as beclin1 and Bcl-2 (52, 53). Proteins in the autophagy pathway can control the switching of cell death between apoptosis and necroptosis (54). Recent advances have illuminated the important involvement of MLKL in diverse cellular processes (Figure 1b) pertaining to membrane trafficking, including autophagy (55, 56), endosomal trafficking, and EV generation (57). Moreover, autophagy can negatively regulate and/or promote pyroptosis and the release of inflammatory cytokines, depending on the cellular context (58). For example, macrophage-derived EVs shuttle HMGB1 via endocytosis and promote hepatocyte pyroptosis by activating the NLRP3 inflammasome (59).

In summary, the regulatory mechanisms for maintaining the intricate balance between cell prosurvival and death pathways in the liver are complex. It is assumed that apoptosis may occur in the early stages of ALD but not in the more advanced inflammatory stages, when necrotic/necroptotic cell death likely fuels inflammation in AH. After the transition to AH, coincident with endotoxemia/bacteremia, pyroptosis may dominate as a form of hepatocellular death, contributing to the recruitment of polymorphonuclear neutrophils and further promoting inflammation. It is also likely that different types of PCD may coexist at each stage of ALD, depending on the specific microenvironment.

The question of whether there is an optimal time point for intervening in a specific pathway of cell death in the progression of ALD is of translational importance. Given the evidence for the overlapping and cell-specific pathways of PCD in murine models of ALD, it will be critical for future studies to comprehensively explore the contribution of PCD, in specific cell types and at different stages of disease, to expand our understanding of the precise role of PCD in the pathogenesis of ALD.

## HEPATIC IMMUNE RESPONSES

### Innate and Adaptive Immune Cells

In ALD, the immune system plays two critical roles throughout the body: removing foreign, gut-derived microbial byproducts via PAMPs and responding to tissue damage and cell death via DAMPs. The immune system responds with a combination of pro- and anti-inflammatory signals within the tissue that lead to clearance of pathogens and dead cells, infiltration of peripheral cells, and resolution of inflammation.

Most research efforts over the last few decades have focused on the molecular and cellular responses that are perturbed directly by alcohol and/or are altered in ALD. For example, binge alcohol consumption causes leakage of gut-derived LPS, leading to TLR4 signaling in liver macrophages, including both resident KCs and monocytes (6). In ALD, macrophages are hypersensitive to LPS, leading to increased inflammatory responses. In recent years, a greater appreciation has developed for a more diverse set of molecular signals and cell types involved in disease progression. Moreover, single-cell RNA-sequencing (scRNA-seq) studies are allowing us to better understand the greater diversity of immune cells in the liver and how they are individually regulated in progression of ALD.

**Figure 2:**
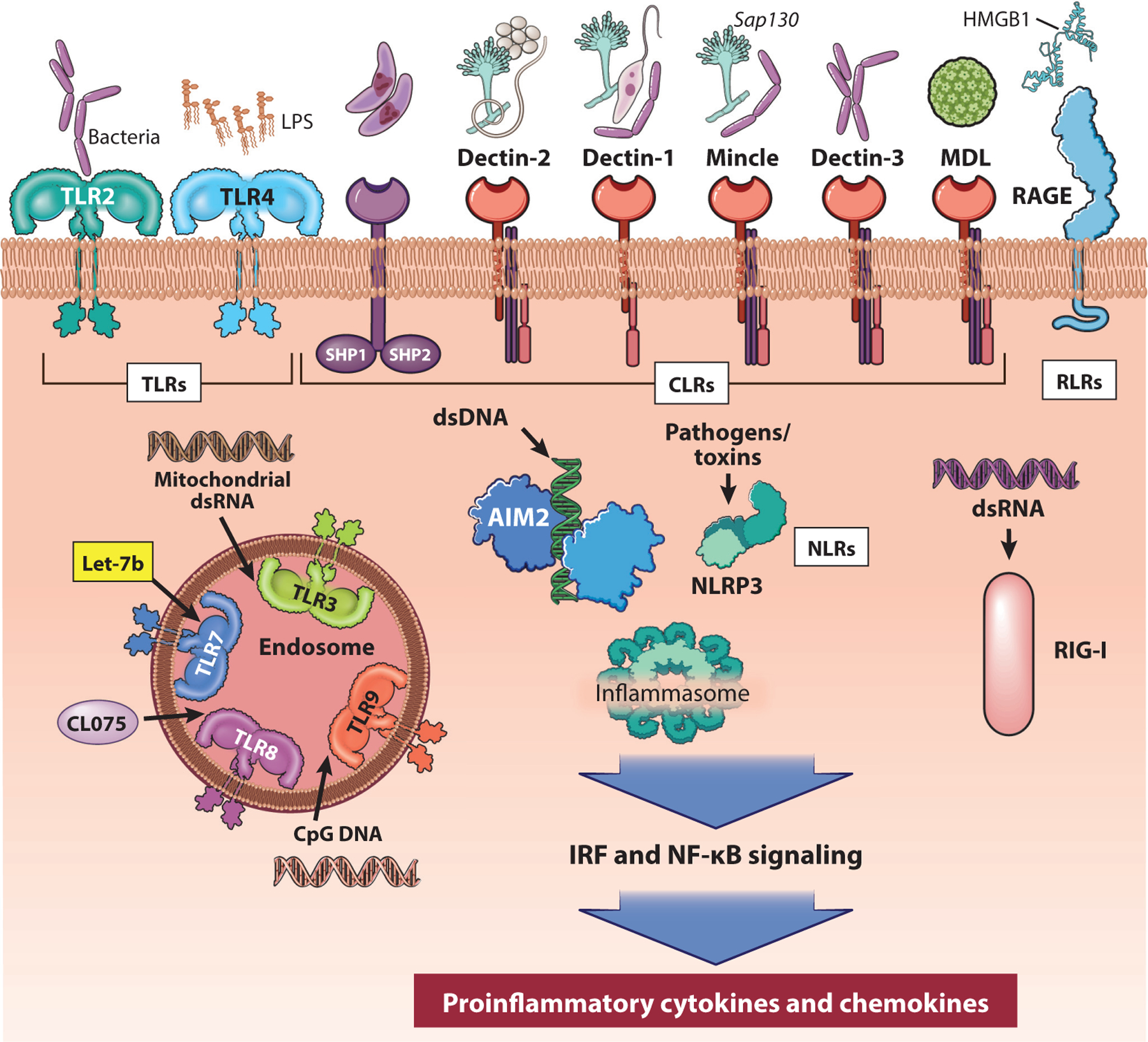
PRR family in ALD. PAMPs and DAMPs signal through PRRs to activate the immune system. Multiple PRRs have been implicated in progression of ALD, including extracellular receptors, such as TLR2/4, CLRs, and RAGE, as well as intracellular receptors including TLR3/7/8/9, AIM2, NLR3, and RLRs. The CLR family senses a broad repertoire of PAMPs, including many diverse fungi, viruses, commensal bacteria, eukaryotic pathogens, and DAMPs that originate from distinct cells and tissues. Large blue arrows indicate the signal transduction cascade. Abbreviations: AIM2, absent in melanoma 2; ALD, alcohol-associated liver disease; CLR, C-type lectin receptor; DAMP, damage-associated molecular pattern; NLR3, nucleotide oligomerization domain (NOD)-like receptor 3; PAMP, pathogen-associated molecular pattern; PRR, pattern recognition receptor; RAGE, receptor for advanced glycation end products; RLR, retinoic acid-inducible gene I (RIG-I)-like receptor; TLR2/4, Toll-like receptor 2/4. Adapted with permission, Cleveland Clinic Foundation ©2022. All rights reserved.

Detection of PAMPs and DAMPs is an essential step in the pathogenesis of ALD in both rodent models and human studies. PAMPs and DAMPs signal through PRRs such as TLR4 to activate the immune system. Many PRRs play essential roles in ALD. PRRs can be classified by the cellular localization, where PRRs at the cell surface detect bacterial and fungal byproducts and DAMPs while intracellular PRRs sense microbial and viral DNAs and RNAs and some host-derived nucleic acids. Often, though, multiple pathways are activated sequentially. As a result, almost every PRR has been implicated in progression of ALD (6, 60) (Figure 2), including intracellular receptors such as TLR3/7/8/9, cGAS, AIM2, NLRs, and extracellular receptors such as TLR2/4 and the C-type lectin receptors (CLRs).

The CLRs have become particularly interesting because they sense a much broader repertoire of PAMPs, compared with the TLR family, including many diverse fungi, viruses, commensal bacteria, eukaryotic pathogens, and DAMPs that originate from distinct cells and tissues. Many of these CLRs are upregulated in ALD, in peripheral blood mononuclear cells from AH patients (61), in livers from patients with ALD (A. Kim & L.E. Nagy, unpublished observations), and in rodent models of ALD (62, 63). Dectin-1, a CLR that detects the pathogenic fungus *Candida albicans*, is upregulated in patients with ALD and increases liver inflammation in response to gut-derived *C. albicans* β-glucans (63). Other CLRs, including mincle, dectin-2, and dectin-3, are upregulated in response to LPS (61). By upregulating CLRs in response to TLR4 signaling, this secondary immune surveillance pathway makes monocytes more sensitive to a broader range of PAMPs and DAMPs, which contribute to inflammation and monocyte infiltration into tissues where damaged cells and foreign particles are found. Both *Mincle* and *Dectin-1* knockout mice are protected from ethanol-induced liver injury in murine models of ALD (62–64).

Activation of PRR signaling leads to cytokine and chemokine expression. Expression of cytokines and chemokines is often exacerbated in ALD and contributes to significant additional tissue damage. Neutrophils, which are recruited by specific inflammatory cytokines and chemokines, play a controversial role in disease progression, as they can help remove dead and dying cells as well as contribute to tissue damage. Other immune cells also respond to these inflammatory signals, including natural killer (NK) cells and T cells. Interestingly, in ALD, NK cells and T cells are thought to have reduced functions and cellular activity, despite increased numbers in the liver, though peripheral cell numbers are decreased and patients manifest lymphopenia (65).

Much of the work discussed so far is rooted in studies from rodent models of ALD, but in patients, ALD is a diverse spectrum of diseases where immune cells have different roles in different stages. Unfortunately, rodent models are unable to replicate certain aspects of AH and alcohol-associated cirrhosis. In addition to species differences, rodent models are unable to account for differences in human diversity, including factors such as sex, genetics, environment, and diet. Thus, much of the field is looking to harness the power of omics technologies to better understand multifactorial aspects of ALD by leveraging access to patient samples. For example, scRNA-seq and bulk RNA-seq technologies have already proven to be useful for understanding the role of immune cells in ALD and AH (61, 66, 67). A recent scRNA-seq study found peripheral monocytes from AH patients to be less functionally diverse than those from HCs. For example, in response to LPS challenge, different monocyte subpopulations in HCs responded with diverse pro- and anti-inflammatory pathways, but in patients with AH, all monocytes were proinflammatory (61). Because ALD and especially AH have increased peripheral and liver inflammation, many therapies currently in development focus on decreasing inflammation. Future studies should consider the diversity of innate immune cell responses to specific challenges.

### Cytokines and Chemokines

Multiple factors and processes that contribute to the progression of ALD are mediated by low-molecular-weight polypeptides known as cytokines and chemokines, produced and released by various cells including liver cells (6, 60).

#### Cytokines.

In patients with ALD and animals exposed to chronic ethanol feeding, a variety of cytokines are reported to be elevated including TNF-α, various ILs (such as IL-1, IL-4, IL-6, IL-10, IL-12, IL-17, and IL-22), and IFN-γ, as well as high-sensitivity C-reactive protein, transforming growth factor beta (TGF-β), and adiponectin. Most of them play dual functions in pathogenesis of ALD (6, 60).

TNF-α is a critical proinflammatory cytokine in ALD (6, 60). Chronic ethanol exposure results in the translocation of LPS from the gut to activate KCs via TLR4. Enhanced production of proinflammatory cytokines, including IL-1 and TNF-α, thereby contributes to hepatocyte dysfunction and PCD, as well as activation of hepatic stellate cells to generate extracellular matrix (ECM) proteins leading to fibrosis/cirrhosis (68). For example, *Tnf-α* knockout mice, mice deficient in different components of the IL-1 pathway, and mice treated with IL-1 receptor antagonist to neutralize the activity of IL-1 are all protected from ethanol-induced liver injury (69). Other cytokines, in particular TGF-β, are also associated with the activation of hepatic stellate cells and collagen production, contributing to the development of fibrosis in patients with ALD (70).

IL-6 is another pleiotropic cytokine that exerts a dual role in liver homeostasis. Elevated IL-6 can reduce liver injury and inflammation through activation of STAT3 and participate in mitochondrial DNA repair in chronic alcohol-fed animals and in patients with alcohol-use disorder with or without liver disease (6, 60). On the other hand, IL-6 promotes human Th17 differentiation and IL-17 production, therefore contributing to ethanol-induced liver inflammation via enhanced recruitment of neutrophils (6, 60).

Interestingly, IL-10, an anti-inflammatory cytokine, known for its hepatoprotective effects, is secreted simultaneously with proinflammatory cytokines. When *Il-10* knockout mice are exposed to chronic ethanol, they exhibit increased inflammatory responses in the liver, associated with increased IL-6/STAT3 activation, but less steatosis and lower serum aspartate aminotransferase and alanine aminotransferase enzyme activity (71).

IL-22, another member of the IL-10 family of cytokines, is also associated with protecting the liver from ethanol-induced injury. In the Gao-binge murine model of ALD, treatment with IL-22 recombinant protein activates hepatic STAT3 and protects mice from hepatic oxidative stress and hepatocyte injury. Importantly, in a human phase II study using a recombinant IL-22 fusion protein (F-652), clinicians observed improved clinical scores as well as decreased liver injury markers when patients with ALD were treated with recombinant IL-22 (72, 73).

#### Chemokines.

A plethora of chemokines, including Gro-α/CXCL1, PF-4/CXCL4, CXCL5, CXCL6, IL-8/CXCL8, CXCL10, CCL2, and CCL20, are positively correlated with higher mortality in patients with AH (74). Among them, hepatic CXCL8/IL-8, a critical and highly upregulated chemokine, is specifically associated with recruiting neutrophils to the liver. IL-8 is induced by TNF-α and by TLR-dependent activation of nuclear factor kappa B (NF-κB). In murine models of ALD, blockade of IL-8 receptors (CXCR1/2) with pepducin antagonist protects mice from liver injury (75, 76). Among the CC chemokines, CCL20, ligand for CCR6, is one of the most upregulated chemokines in liver samples from patients with AH. Expression of CCL20 is induced by many inflammatory mediators, such as LPS, TNF-α, and IL-1β, and regulates liver inflammation and fibrosis by acting as a chemoattractant for lymphocytes and neutrophils (77). Higher concentrations of macrophage migration inhibitory factor (MIF), another pleiotropic cytokine/chemokine, in the suprahepatic circulation are associated with higher mortality in AH patients. Interestingly, hepatocyte-derived MIF contributes to the upregulation of a series of chemokines, including *Cxcl1, Cxcl5*, *Cxcl6*, *Cxcl*8, *Ccl2*, and *Ccl20*, in livers of mice exposed to Gao-binge acute-on-chronic ethanol feeding (78). The coordinate expression of chemokines suggests that therapeutic targets for upstream signals in the chemokine expression pathway may be useful therapeutic targets for treatment and/or prevention of ALD.

### Complement

Complement comprises a system of more than 40 plasma and membrane-associated proteins (79) consisting of activation components, regulatory factors, and receptors. Complement is part of the innate system and provides links to adaptive immunity. It plays a vital role in host response to microbial infection and in the response to tissue injury, thus playing a vital role in rapid immune responses, as well as maintaining multiple metabolic responses, including lipid metabolism and wound healing. Hepatocytes are the primary source for circulating complement factors, although growing evidence points to the importance of local complement production by a number of cell types (79, 80).

The system is activated via the classical pathway (CP), the lectin pathway (LP), or the alternative pathway (AP). The CP, involving the C1 complex (C1q, C1r, and C1s), C2, C3, and C4 components, is activated by antigen-antibody immune complexes binding to C1. The LP, though functionally similar to the CP, differs in its mode of activation. It is activated by the binding of lectins or ficolins to carbohydrate ligands on pathogens. The AP, on the other hand, is not activated by exogenous materials but is constantly activated at low levels through spontaneous plasma C3 hydrolysis, and it involves complement factors B, D, H, I, and P. Activation of these three pathways converges at the terminal pathway with the formation of C3 and C5 convertases, subsequently generating the main effector molecules with the eventual formation of the membrane attack complex, C5b-9 (79).

Mounting evidence in murine models has implicated complement in the initiation and development of ALD. Mice deficient in *C3* or *C5* are protected from chronic ethanol-induced liver injury, while mice deficient in *CD55*, a complement regulator, have exacerbated injury (81). Specific inhibition of C3 activation with complement receptor 2 (CR2)-Crry significantly decreased inflammatory responses and hepatic steatosis in mice exposed to ethanol (82). One mechanism for complement activation in response to ethanol was found to be via binding of C1q to apoptotic hepatocytes, causing an initial rise in inflammatory cytokine expression (83). Mice with a *C1qa* deficiency or treated with *Cinryze* (C1-INH), a purified C1 inhibitor, are protected from chronic ethanol (84). Taken together, these studies suggest that activation of complement via the CP contributes to ethanol-induced liver injury. However, the role of complement in disease progression is more complex, with recent data demonstrating cell-specific roles for C5aR1 in myeloid and non-myeloid cells in liver and adipose tissue (85, 86). Similarly, the specific pathways of complement activation may also be important. For example, in contrast to the injurious role of CP activation, activity of the AP via factor D (FD) protected mice from chronic ethanol-induced injury (87).

A few studies have also implicated complement in patients with ALD. For example, plasma C3a concentrations are associated with fatty liver and hepatocellular damage in heavy drinkers (88). In another study, C1q, C3, C5, and C5aR immunoreactivities were increased in the liver biopsies of patients with AH compared with HCs; expression of C1q and C5, but not C3, mRNA was also increased in livers of patients with AH (80). In a recent study quantifying complement components in plasma from patients with moderate and severe AH, factors C4b, C4d, CFD, CFI, C5, and sC5b9 could distinguish healthy subjects from patients with AH. Importantly, both CFI and sC5b9 were negatively associated with 90-day mortality in patients with AH (89).

Collectively, the data from murine models of ALD and from patients indicate a complex contribution of complement in ALD and suggest that complement may be useful as a prognostic and diagnostic indicator in patients with AH.

## HEPATIC REGENERATION: ROLE OF CELL-CELL CROSS TALK

The liver is considered the only fully regenerative organ in the human body, but during chronic liver diseases, such as ALD, hepatocyte regeneration is compromised. In a healthy liver, hepatocytes have different functions depending on their position between the portal triad and the central vein, termed hepatic zonation. Hippo/YAP signaling is required for periportal hepatocyte gene expression. In severe AH, upregulation of YAP, downregulation of ESRP2, and differential splicing of the nuclear receptor HNF4α results in aberrant upregulation of periportal hepatocyte genes, hepatocyte fetal reprogramming, and activation of the ductular reaction, where periportal hepatocytes and liver progenitor cells activate and become either mature hepatocytes or cholangiocytes (67, 90, 91).

Hepatic endothelial cells (ECs), including periportal ECs, liver sinusoidal ECs (LSECs) and periportal ECs, also play a critical role in liver regeneration by direct interaction with macrophages and hepatocytes. While periportal hepatocytes are expanded and central hepatocytes are depleted in liver regeneration, periportal ECs are diminished and central vein ECs are expanded in severe AH (66). ECs can promote inductive angiogenesis through release of angiocrine factors, including hepatocyte growth factor, Wnt2 (92), and Wnt9 (93). Wnt2 is expressed in both central vein and sinusoidal ECs and macrophages, while Wnt9b is specifically expressed in central vein ECs and secondarily in macrophages (93, 94). Pericentral hepatocytes are regulated by WNT signaling, and, in ALD, WNT signaling is also dysregulated. In AH, expression of WNTs and their receptors, the FZD family, varies with disease severity (66). In healthy livers, the predominant genes are WNT2 and FZD4, while in moderate AH, WNT5a and FZD5 are upregulated, which is notable because WNT5a is thought to play a role in active liver regeneration. In severe AH, WNT5a is downregulated, while many other WNTs and FZDs are expressed. Many of these FZD genes have been previously implicated in human hepatocellular carcinoma (HCC) (95). Considering WNT signaling alone, we hypothesize that there is a progression from healthy human liver to moderate AH, where liver regeneration can occur, to severe AH, where regeneration is dysregulated and signs of HCC may start to develop.

## ROLES OF LIVER SINUSOIDAL ENDOTHELIAL CELLS AND STELLATE CELLS

### Liver Sinusoidal Endothelial Cell Dysfunction

The liver filters toxins through sinusoidal channels lined with KCs. This is mediated by LSECs that, when exposed to blood from the gut and systemic circulation, remove and recycle blood-borne proteins and lipids through the presence of highly permeable fenestrae (96). The lack of a basement membrane contributes to the permissive nature of sinusoidal endothelium (96), allowing the LSECs and KCs to endocytically take up and eliminate invading pathogens (97). Dynamic changes in hepatic fenestration number and size are highly regulated, with vascular endothelial growth factor (VEGF) implicated as essential in this regulation (97). Alcohol and dietary constituents can also modulate fenestration function by changing the access of macro-molecules to parenchymal cells and by allowing circulating viruses to infect hepatocytes (98). LSECs maintain a balance between tolerance and effector immune responsiveness, facilitated by their innate and adaptive immunological functions (96). Defenestration and LSEC activation occur early in animal models of fatty liver disease (99), contributing to the activation of hepatic stellate cells (HSCs) and fibrogenesis. Capillarization also increases hedgehog signaling (100) and impairs VEGF-dependent endothelial nitric oxide (NO) synthase activity (97). Interestingly, LSECs also promote reversion from activated HSCs to a quiescent phenotype via NO production (101). LSECs undergo many changes during injury that promote the recruitment of proinflammatory immune cells including increased expression of adhesion molecules such as intercellular adhesion molecule 1, vascular adhesion protein 1, and stabilin 1, which promote T and B cell adhesion; chemokines such as CXCL16, CXCL9, and CX_3_C-chemokine ligand 1, which contribute to adhesion of transmigrating T cells and monocytes; and hyaluronan, which promotes neutrophil adhesion (96).

### Hepatic Stellate Cell Activation

Long-term liver fibrosis leads to accumulation of ECM proteins and results in the replacement of parenchyma with nonfunctional scar tissue. HSCs are the cells predominately responsible for the production of ECM in the fibrogenic process. HSCs, located in the space of Disse, are surrounded by hepatocytes and LSECs (102). HSCs secrete laminin, proteoglycans, and collagen IV to form basement membrane structures and remain in a quiescent state until activated. Activated and pro-liferating HSCs express α-smooth actin and upregulate the synthesis of type I and III collagens and ECM proteins such as fibronectin (103). HSC activation is promoted by a number of factors present within the hepatic microenvironment during chronic ethanol exposure. For example, HSCs are activated by apoptotic hepatocytes as well as multiple paracrine signals from neighboring cells including KCs, LSECs, platelets, and infiltrating immune cells. KCs produce cytokines such as TGF-β, TNF-α, and IL-1 that stimulate proliferation of HSCs (103). HSCs also respond to ROS released from neutrophils, and inflammatory lipid peroxides from damaged hepatocytes during progression of liver disease (102, 104) and complement C5a stimulate HSC chemotaxis and migration (105).

### microRNAs: Function at All Stages of Liver Injury

miRNAs play a critical role in mediating steatosis, inflammation, injury, and gut permeability in the pathogenesis of ALD. Ethanol and its metabolites induce miRNA dysregulation in a variety of organ systems and circulation. Using RNA sequencing, global changes in miRNA regulation have been identified that are associated with polarization phenotypes in KCs from rats after chronic ethanol and in peripheral blood mononuclear cells from patients with AH. These polarization-associated miRNAs are localized to coordinately regulated clusters. For example, miR-125a-5p, miR-125a-3p, and miR-99b-5p and the host gene sperm acrosome associated protein 6 (SPACA6) were upregulated in AH patients (106). In the liver, miR-122 in hepatocytes regulates steatosis (107, 108), while miR-155 in KCs yields a proinflammatory state, perpetuating liver injury (107). miR-212 has also been implicated in the alcohol-induced impairment of gut barrier function (109). Additionally, ALD is associated with an increase in hepatocellular death through death receptor ligands regulated in part by ubiquitination enzymes (E1–E3) that control protein degradation and localization. Recent work implicated miR-150–5p as affecting Fas-associated death domain (FADD) degradation by inhibiting cytokine-inducible SH2-containing protein (CISH) expression. In addition, miR-150–5p is elevated in livers of patients with AH and mice after Gao-binge ethanol exposure, suggesting a role in the pathogenesis of ALD.

miRNA profiles of human HSCs change during activation in cell culture (110), and modulatory roles of miRNAs in the expression of liver fibrosis-associated genes have been reported (111). Several miRNAs are classified as antifibrotic such as miR-19b, miR-34a-5p, miR-146a, miR-133, miR-23b-27, and miR-134 (112, 113). Overexpression of miR-133 in HSCs inhibits collagen expression and downregulation during fibrogenesis and TGF-β treatment (112). Profibrogenic miRNAs include miR-942 and miR-125b (111, 114). Codelivery of miR-29b1 with a hedgehog inhibitor can decrease collagen and α-SMA (113). Upregulated in fibrosis, miR-542–3p was found to control the activation of HSCs and promote liver fibrosis by downregulating BMP-7 expression. Overexpression of miR-199 and miR-200 families was correlated with the TGF-β/SMAD signaling pathway during liver fibrogenesis in both a fibrotic mouse model and human clinical samples of patients with fibrosis (115). Transfection of miR-129–5p mimic reduced the expression of type I collagen in activated HSCs and was associated with a reduction in fibrotic injury (116).

## ORGAN-ORGAN INTERACTIONS

### Gut-Liver Axis

**Figure 3:**
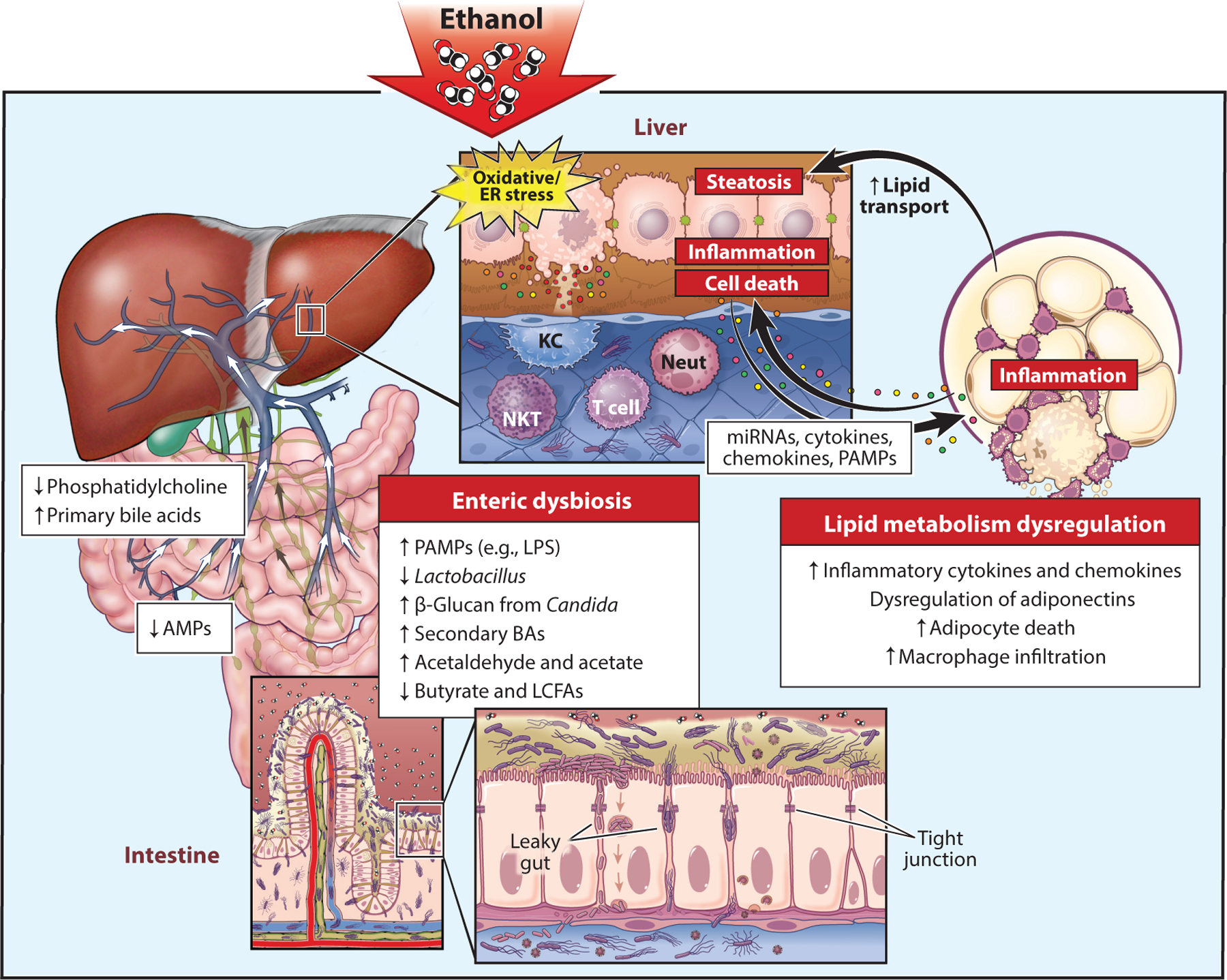
Organ-organ interactions in the pathogenesis of ALD. The gut communicates with the liver through the portal vein, biliary system, and other circulating soluble mediators. Chronic alcohol consumption results in enteric dysbiosis and bacterial overgrowth. ALD-associated dysbiosis is characterized by a reduction in *Lactobacillus* and *Candida* overgrowth. Bacterial overgrowth causes an increase in secondary BAs, resulting in an overall increase in hepatic BA synthesis. A reduction in hepatic phosphatidylcholine is also seen in ALD, causing triglyceride accumulation in the liver. Alcohol-associated dysbiosis in mice was further linked to reduced LCFA biosynthesis and short-chain fatty acids, including butyrate. Ethanol and its metabolite acetaldehyde have been implicated in weakening the intestinal tight junctions and inflammatory response by downregulating AMPs in the intestine. Consequently, increased translocation of PAMPs and gut metabolites elicits intestinal and hepatic inflammatory responses, leading to progressive liver damage. Ethanol also causes adipocyte death and metabolic and immune dysfunctions of adipose tissue. Adipose-liver cross talk is mediated by the release of mediators, including neurotransmitters, cytokines, chemokines, adipocytokines, miRNAs, extracellular vesicles, and metabolites. Abbreviations: ALD, alcohol-associated liver disease; AMP, antimicrobial peptide; BA, bile acid; ER, endoplasmic reticulum; KC, Kupffer cell; LCFA, long-chain fatty acid; LPS, lipopolysaccharide; miRNA, microRNA; Neut, neutrophil; NKT, natural killer T cell; PAMP, pathogen-associated molecular pattern. Adapted with permission, Cleveland Clinic Foundation ©2022. All rights reserved.

The liver and intestine communicate via the portal vein, biliary system, and other circulating soluble mediators (117). Therefore, the liver is the first organ exposed to gut-derived microbial components and metabolites. Alcohol consumption influences multiple aspects of gut physiology, specifically increasing gut permeability as well as affecting microbial composition and metabolism (117). Most long-term heavy drinkers develop steatosis, but only 10–20% develop progressive liver disease (6). Current opinion indicates that, in addition to the direct effects of alcohol on the liver, altered gut physiology contributes to the progression of ALD (118) (Figure 3).

#### Dysbiosis.

Alcohol intake causes a striking reduction in fungal and bacterial diversity. Dysbiosis is not only a consequence of alcohol intake but also regulates the individual susceptibility and severity of ALD (118). Commensal fungi are reduced while there is an overgrowth of *Candida* species (63). Similarly, there is decreased abundance of beneficial bacteria such as Ruminococcaceae, *Faecalibacterium*, and *Prevotella*, with a concomitant increase in gram-negative bacteria, for example, Proteobacteria, Enterobacteriaceae, and *Escherichia*. Most of the beneficial bacteria produce short-chain fatty acids, reported to maintain and improve gut health (117). The dysbiotic pressure leads to production of virulence factors by certain bacteria. For example, cytolysin, produced by *Enterococcus faecalis*, directly affects hepatocyte survival. While there is no overall increase in the number of *E. faecalis*, the presence of cytolysin is highly correlated with disease severity in patients with AH (119).

#### Loss of gut integrity.

Gut permeability increases with ethanol consumption (120) and is a prerequisite for development of ALD in murine models (121). Alcohol consumption reduces mRNA of several junctional proteins such as occludin, zonula occludens-1, and claudins 3 and 4 (120). A loss of permeability contributes to translocation of microbes and microbial components into the systemic circulation. Importantly, the epithelial barrier has additional defenses, including a thick mucilaginous layer composed mainly of mucin (Muc) 2 produced by goblet cells, and secretes various antimicrobial peptides (AMPs). Chronic ethanol has complex effects on these defenses. For example, ethanol increases Muc-2 levels in patients with ALD, and *Muc2* deficiency protects mice from chronic ethanol-induced injury. In contrast, chronic ethanol reduces expression of C-type lectin AMPs Reg3β and Reg3γ, and overexpression of Reg3γ protects from injury (122).

#### Translocation of PAMPs, viable microbes, and microbial metabolites.

As discussed above, loss of gut integrity and bacterial dysbiosis contribute to increased exposure of the liver to PAMPs. In addition, viable bacteria can also cross the damaged gut epithelial barrier, contributing to liver injury (119, 123). There is also a growing appreciation that many microbial metabolites are detectable in the portal and systemic circulation in response to ethanol; some of these metabolites likely contribute to ethanol-induced injury. For example, ethanol-mediated gut dysbiosis reduces the short-chain fatty acid (SCFA) composition of the gut (124), thereby affecting colonocyte and enterocyte survival and maintenance of gut barrier. This causes loss of the anti-inflammatory effect of SCFAs in the gut (125). In patients with advanced cirrhosis, low circulating levels of butyrate correlate with increased proinflammatory markers and serum endotoxin (126). The gut microbial metabolite trimethylamine is also elevated in patients with AH and contributes to chronic ethanol-induced liver injury in murine models (127).

#### Bile acids.

Ethanol intake alters qualitative and quantitative bile acid composition in both the liver and gut. Gut bacteria deconjugate primary bile acids via bile salt hydrolase. Deconjugation prevents their reabsorption, thereby maintaining bile acid homeostasis. Chronic ethanol administration increases hepatic bile acid synthesis as well as plasma and fecal concentrations of unconjugated bile acids (128). In patients with AH, bile acid homeostasis and its associated signaling is dysregulated (129). For example, farnesoid X receptor (FXR) signaling, an essential part of the negative feedback mechanism regulating hepatic bile acid synthesis, as well as glucose and lipid metabolism, is reduced in patients with ALD (130).

### Adipose-Liver Axis

While changes in adipose tissue function are traditionally associated with nonalcoholic-associated liver disease, considerable evidence indicates that chronic alcohol also impairs the function of adipose tissue. Chronic ethanol is associated is associated with impaired metabolic, endocrine, and immune functions of adipose tissue, changes that likely contribute to the progression of ALD (131, 132) (Figure 3).

#### Metabolic regulation.

Chronic alcohol exposure increases lipolytic activity in adipose tissue, thereby increasing circulating nonesterified fatty acids (NEFAs) and increasing the exposure of the liver NEFAs, where they are esterified and contribute to hepatic steatosis (133). Saturated fatty acids have a greater hepatotoxic effect and trigger hepatocyte apoptosis through activation of the c-Jun N-terminal kinase pathway (134). Saturated NEFAs also exert proinflammatory effects through the NF-κB pathway and activation of KCs (135), thereby furthering ALD.

#### Endocrine regulation.

Alcohol abuse increases circulating levels of leptin, visfatin, and chemerin, causing exacerbated fibrotic response, proinflammatory cytokine production from myeloid cells, and immune cell infiltration (132), respectively. Provision of exogenous adiponectin to mice protects from ethanol-induced liver injury (136); however, the relevance of these data to human ALD is not known.

#### Immune regulation.

Chronic ethanol exposure increases inflammatory responses in adipose tissue. Interestingly, adipocytes express CYP2E1 in response to chronic ethanol, likely contributing to oxidative stress and adipocyte cell death, characterized by crown structures (131). Dying adipocytes recruit complement C1q to facilitate clearance, but in the context of ethanol, this response leads to activation of complement and generation of anaphylatoxins (86, 131, 137). Complement activation in turn leads to increased expression of inflammatory cytokines and contributes to impaired regulation of lipid metabolism (131, 132).

Of translational interest, the interaction between ethanol and obesity is particularly important, as epidemiological data suggest that obesity and metabolic syndrome exacerbate progression of ALD (138–140). Importantly, for treatment of heavy drinkers, cessation of drinking rapidly normalizes adipose tissue function (141).

## POTENTIAL THERAPEUTIC TARGETS AND CLINICAL TRIALS FOR ALD

### Targeting Hepatocyte Injury

**Figure 4:**
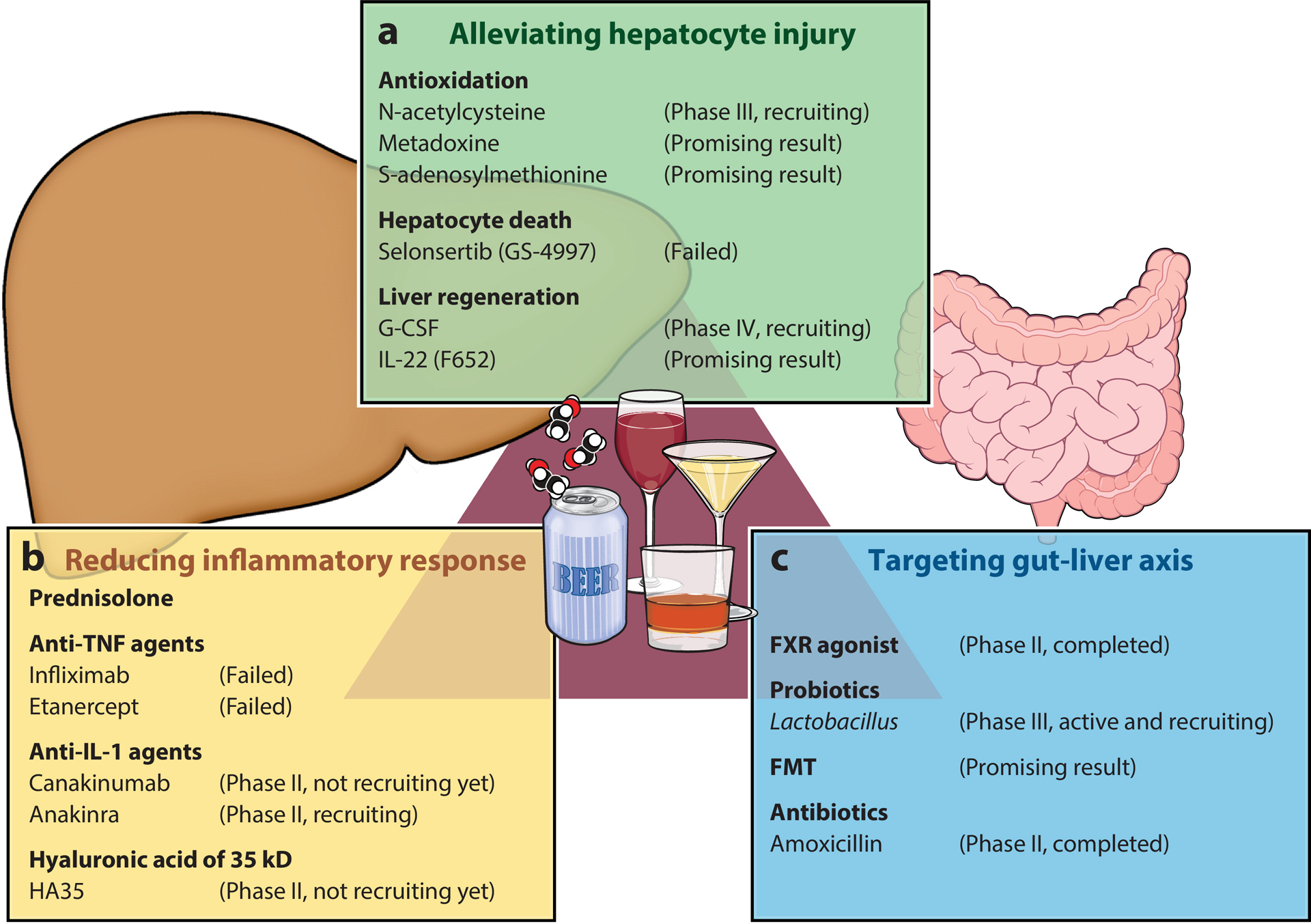
Therapeutic targets and clinical trials for alcohol-associated liver disease. Three approaches are currently being investigated: (*a*) strategies to reduce hepatic injury by decreasing oxidative stress, decreasing death of hepatocytes, and improving hepatic regeneration; (*b*) use of agents to reduce inflammation, including the classic agent prednisolone, as well as more specific targets including TNF and IL-1; and (*c*) targeting gut health via treatment with probiotics or fecal transplants and FXR agonists. Abbreviations: FMT, fecal microbiota transplantation; FXR, farnesoid X receptor; G-CSF, granulocyte colony stimulating factor; IL, interleukin; TNF, tumor necrosis factor. Adapted with permission, Cleveland Clinic Foundation ©2022. All rights reserved.

Accumulating evidence suggests that hepatocyte injury resulting, at least in part, from ethanol-induced oxidative stress and innate immune responses plays a crucial role in progression of ALD (60). Thus, protecting hepatocytes from injury is viewed as a potential therapeutic strategy (Figure 4). Chronic exposure to ethanol induces glutathione depletion, which makes hepatocytes more vulnerable to oxidative stress (142). Oxidative stress is one of the key mechanisms leading to hepatocyte injury in ALD; however, classical antioxidant molecules alone (N-acetylcysteine or metadoxine) are not effective in severe forms of AH (143, 144). One of the reasons for the failure of these antioxidant therapies in AH may be the lack of specific mitochondrial antioxidant effect. Colell and colleagues (145) reported that S-adenosylmethionine could be a potential therapeutic option for ALD, because this molecule restores glutathione in the mitochondria and improves steatosis in rodents. More clinical studies are required to clarify the benefits of mitochondrial-targeted antioxidants for the treatment of AH.

Hepatocyte death is another promising therapeutic target for ALD. As reviewed above, multiple pathways are associated with ALD (146). However, currently there is a lack of therapeutic agents that target these modes of hepatocyte death. In addition, since multiple forms of cell death are associated with ALD, inhibiting individual cell death pathways may be insufficient to improve AH. In a phase II clinical trial, selonsertib (GS-4997), an oral inhibitor of apoptosis signal regulating kinase-1 (ASK-1) enzyme, combined with prednisolone showed no advantage over prednisolone alone in the treatment of severe AH (NCT02854631).

Due to the difficulty in inhibiting hepatocyte death, promoting liver regeneration is considered a complementary therapeutic strategy. The granulocyte colony stimulating factor (G-CSF), a potent growth factor, has been proposed to promote hepatocyte regeneration in severe AH. Results of a meta-analysis suggest that G-CSF is associated with a reduction in mortality by more than 70% at 90 days in patients with AH (147). However, due to the heterogeneity of clinical studies in Asia and Europe, the therapeutic effects of G-CSF need to be interpreted with caution. IL-22, one of the major cytokines of the anti-inflammatory family, provides liver protection and promotes regeneration. Currently, a phase II open-label clinical trial is investigating the impact of an IL-22 agonist (F-652) on AH patients [MELD (model for end-stage liver disease) scores of 11–28]. F-652, which has the same mechanism of action as the native IL-22, is a recombinant fusion protein of human IL-22 and human IgG2 fragments. F-652 associated with a high rate of improvement, as determined by MELD and Lille scores, increases in markers of hepatic regeneration, and reductions in markers of inflammation (73).

### Reducing Inflammatory Responses

Chronic inflammation is a critical factor in the development of ALD (148), suggesting that modulating the inflammatory response is a promising therapeutic strategy for the improvement of ALD (Figure 3). Glucocorticoids (e.g., prednisolone) are currently commonly used as first-line anti-inflammatory agents in patients with severe AH; however, prednisolone is ineffective in most patients and increases the risk for bacterial and fungal infections. Since many immune cells (KCs, neutrophils, NK cells, etc.) and inflammatory mediators (TNF-α, TLR4, IL-1β, etc.) play a dual role in liver injury and liver regeneration, it is necessary to consider comprehensive treatment strategy, rather than simply suppressing or promoting inflammatory responses. Two randomized controlled studies with anti-TNF agents (infliximab and etanercept) were negative, with more deaths occurring in the anti-TNF arm (149, 150). Two ongoing randomized clinical trials are investigating the impact of anti-IL-1 on AH patients. The first trial, being conducted in the United Kingdom, is testing the efficacy and safety of IL-1β antibody (canakinumab) in patients with severe AH [modified Maddrey’s discriminant function (mDF) ≥ 32]. The primary end point is histological improvement of AH on liver biopsy after 28 days of treatment (NCT03775109). The other study, based in the United States, is mainly evaluating the effects of IL-1 receptor antagonist (anakinra) on 90-day mortality in patients with severe AH (NCT04072822). On the basis of the role of TLR4 in the pathophysiology of ALD, TLR4 antagonists seem to be promising candidates for the treatment of AH. Hyaluronic acid of 35 kD (HA35), a small and specific-sized molecule of hyaluronic acid, inhibits the ethanol-induced TLR4 signaling pathway in KCs in mouse models (151). A randomized controlled trial (RCT) on the effects of HA35 on the change of skeletal muscle mass in patients with AH is registered, but patient recruitment has not started (NCT05018481). FXR agonists provide hepatoprotective effects by exerting anti-inflammatory and antioxidant effects and by regulating lipid and bile acid metabolism. A phase II randomized clinical trial using obeticholic acid, an FXR agonist, in patients with AH (MELD scores of 12–19) was conducted; however, the clinical trial was terminated because of hepatotoxicity associated with obeticholic acid (NCT02039219).

Inhibition of the activation of inflammatory cells and the release of inflammatory mediators via the reduction of LPS had positive effects for the improvement of ALD in animal models. The ability of antioxidant agents, HA35, and the microbiome-based therapies to reduce liver damage by preventing LPS flux from the intestinal tract has been demonstrated in animal models (152), but further clinical studies are needed to validate these effects.

### Microbiome-Based Therapies

In recent years, as the awareness of the impact of ethanol on the gut pathophysiology has increased, the gut microbiota have become one of the major targets in the development of therapeutics for ALD (Figure 3).

Accumulating evidence from early-stage clinical studies shows interesting results. For example, Han and colleagues (153) reported the importance of probiotics in a multicenter RCT, with the probiotic group having significantly reduced TNF-α (*p* = 0.042) and LPS (*p* = 0.028) compared with the placebo group. Two ongoing randomized clinical trials are investigating the impact of probiotics on AH patients. The first trial, being conducted in the United States, is testing the efficacy and safety of *Lactobacillus rhamnosus* GG in patients with moderate AH (MELD score <21). The primary end point is the change in MELD score after 30 days (NCT01922895). The other study, based in Korea, is evaluating the effects of *Lactobacillus rhamnosus* R0011 and acidophilus R0052 on liver enzyme, endotoxin, and cytokine levels after 7 days in 140 AH patients (NCT02335632). Antibiotics can also alter the gut microbiota. However, hepatitis and systemic inflammation were not improved after 7 days of antibiotic cocktail using vancomycin, gentamicin, and meropenem (9). A multicenter, double-blind RCT evaluating the combination effect of corticosteroids and the antibiotic amoxicillin in severe AH has been completed in France (NCT02281929), and the results need to be confirmed. For now, the role of routine antibiotics in the management of AH has yet to be established.

Fecal microbiota transplantation (FMT) may be a solution for restoring healthy gut flora. Philips and colleagues (154, 155) reported in a pilot study and subsequent open-label trial that FMT in patients with severe AH from healthy donors improved survival and liver function due to reduced gut bacteria that contribute to the development of AH. The first report showed that daily 7-day administration of FMT through a nasoduodenal tube significantly improved 1-year survival (87.5% versus 33.3%, *p* = 0.018) in patients with steroid-resistant AH (*n* = 8) compared with a control group using a standard surgical seat (*n* = 18) (154). Furthermore, they analyzed the microbiota in stool and reported the coexistence of donor and recipient species at 6–12 months after FMT, suggesting that FMT alters the recipient’s gut microbiota network and that these changes are sustained over time. In their second study, the prognostic impact of FMT was tested in a cohort of 51 male patients with severe AH. Patients were divided into four treatment groups: FMT group (*n* = 16), steroid treatment group (*n* = 8), nutritional support treatment group (*n* = 17), and pentoxifylline treatment group (*n* = 10), and the effects on prognosis were investigated retrospectively. Survival rate after 3 months was significantly better in the FMT treatment group than in the other treatment groups, at 75%, 38%, 30%, and 29%, respectively (*p* = 0.036). Further more, favorable changes in the composition of intestinal microflora were observed (155). These studies suggest that the donor microbiota may modify the recipient microbiota and improve ALD without complications, even in patients with severe AH.

Duan and colleagues (119) recently found that cytolysin-secreting *E. faecalis* strains are an important factor in exacerbating liver cell damage and death in patients with severe alcoholism. The authors found that patients with alcoholism had much higher numbers of *E. faecalis* in their stool than nonalcoholics or patients with alcohol use disorders. Interestingly, the total number of *E. faecalis*, not just the presence of cytolysin-positive strains, may be important in the severity of liver disease and subsequent mortality. Using humanized mice colonized with bacteria collected from the feces of AH patients, the researchers showed that certain bacteriophages specifically target cytolytic *E. faecalis*, reduce cytolysin in the liver, and eliminate ethanol-induced liver disease. Importantly, this approach provides a way to precisely edit the gut microbiota. Clinical trials with a larger number of people are needed to validate these results.

## SUMMARY POINTS

1. The pathology of alcohol-associated liver disease (ALD) is complex, involving direct effects of ethanol and its metabolites on the liver, as well as changes in metabolic homeostasis, oxidative stress, and inflammation.
2. Multiple cell-cell and organ-organ interactions are now understood to contribute to the progression of ALD.
3. Importantly, basic and translational studies have led to the identification of effective and rationally designed therapeutic targets.
4. Multiple clinical trials are under way aimed at reducing disease progression and improving the treatment of patients at different stages of ALD.
